# Investigate the relationship between obstructive sleep apnea and cardiac arrhythmia after CABG surgery

**DOI:** 10.1186/s12872-023-03694-x

**Published:** 2024-01-23

**Authors:** Somayeh Mohammadi, Ezzat Paryad, Atefeh Ghanbari Khanghah, Ehsan Kazemnezhad Leili, Marzieh Jahani Sayad Noveiri

**Affiliations:** 1 School of Nursing and Midwifery, Guilan University of Medical Sciences, Rasht, Iran; 2 Department of Nursing, GI Cancer Screening and Prevention Research Center, Guilan University of Medical Sciences, Rasht, Iran; 3 Department of Nursing, Social Determinants of Health Research Center (SDHRC), Guilan University of Medical Sciences, Rasht, Iran; 4 Department of Biostatics, School of Nursing and Midwifery, Guilan University of Medical Sciences, Rasht, Iran; 5 Department of Medical Surgery, School of Nursing and Midwifery, Guilan University of Medical Sciences, Rasht, Iran

**Keywords:** Coronary Artery Bypass Grafting, Sleep apnea, Obstructive, Cardiac dysrhythmia

## Abstract

**Background and objective:**

Heart rhythm disorder is one of the most common problems after coronary artery bypass graft surgery. Various factors, such as the history of sleep apnoea before the operation, may aggravate the occurrence of this disorder. The present study was conducted to determine the relationship between sleep apnoea before surgery and heart rhythm disorder after surgery in patients undergoing coronary Artery Bypass Grafting in 2019.

**Methods:**

This analytical cross-sectional study was conducted on 192 patients who were selected by sequential sampling. The research tool included demographic information, a checklist of heart rhythm disorders, and the Berlin sleep apnoea questionnaire. Descriptive statistics and the Chi-square test, Fisher's exact test, Mann–Whitney’s U-test, and logistic regression were used to analyze the data.

**Results:**

A total of 71.35% of the samples were male, and the mean age of the participants was 57.8 ± 7.5 years. Also, 46.0% of the samples had sleep pane and 21.35% had rhythm disorder. The most frequent heart rhythm disorder in patients with obstructive sleep apnoea was atrial fibrillation. There was a significant relationship between the occurrence of rhythm disorder and a history of smoking (*P* = 0.021), and the regression model showed that a history of smoking is the only variable related to the occurrence of rhythm disorder after coronary Artery Bypass Grafting (*P* = 0.005, CI 95%: 6.566–1.386, OR = 3.017).

**Conclusions:**

The results showed that there is no statistically significant relationship between sleep apnea and rhythm disorder after coronary artery bypass surgery.

## Key messages

**What is already known on this subject?:**

Sleep disorders such as sleep apnea is one of the symptoms that is observed in many patients with coronary artery disease, but contradictory results have been found, a definite conclusion regarding the relationship between this disorder and some complications after it, such as the occurrence of rhythm disorder before and After surgery, it made coronary artery grafting impossible, so the necessity of conducting more studies in this matter has been emphasized.

**What might this study add?:**

The results showed that there is no statistically significant relationship between sleep apnea and the occurrence of rhythm disorder after coronary artery bypass surgery, and the only related and predictive variable with the occurrence of rhythm disorder after coronary artery bypass surgery was the history of smoking.

**How might this impact on clinical practice?:**

Considering the increasing number of coronary patients requiring coronary artery bypass surgery and occupying hospital beds, determining the predictors of complications after surgery can help reduce long hospital stays.

## Introduction

Lifestyle changes, unhealthy eating habits and smoking have increased the incidence of coronary artery disease [1]. Following the increase in the frequency of coronary artery disease, the rate of coronary artery bypass graft surgery (CABG) is also rising. This operation is very costly and filled with complications both for the patient and the health system of countries [2]. A total of 640,000 CABG procedures are performed annually in the United States [3]. Heart rhythm disorders are one of the most important complications of CABG [4]. Cardiac rhythm disorder after heart surgery can be associated with many problems and lead to complications such as increased blood clot formation, kidney problems, and pulmonary embolism [5, 6]. Other complications of heart rhythm disorder include increased length of hospital stay, increased rate of re-hospitalization, reduced ventricular ejection fraction, reduced blood pressure due to rapid ventricular response, heart attack, re-intubation, and higher risk of death, and also the imposition of heavy costs on the patient and health system [7, 8].

Heart rhythm disorders after heart surgery [7] may have several reasons, one of which is obstructive sleep apnoea [9, 10]. Sleep apnoea is divided into two types: Obstructive sleep pane and central sleep apnoea. Respiratory obstruction is related to the upper airway and takes the form of partial or complete obstruction of the air flow, causing hypoxia and ventilatory effort and then waking the person up and opening the airway [11]. The obstructive pane is defined as the absence of airflow for at least 10 s despite an active ventilatory effort accompanied by chest and abdominal movements. The respiratory pause index refers to the mean number of respiratory pauses in one hour of sleep, and if it is more than 30 events per hour, the severity is considered high [9]. To date, conflicting results have been reported on the relationship between sleep apnoea and the occurrence of heart rhythm disorders after heart surgery. The results reported by Mungan et al. in a study conducted to identify the frequency of sleep apnoea in patients who were candidates of CABG showed that 33 of the 73 patients who developed AF after surgery had sleep apnoea [12]. The results of the study by Nunes et al. in Brazil, which was conducted on two groups of patients undergoing heart surgery and abdominal surgery, showed that patients with sleep apnoea are more susceptible to AF [10].

Due to the increasing number of CABGs performed and consequently more occupied hospital beds, the relationship between obstructive sleep apnoea before surgery and the occurrence of cardiac dysrhythmia after heart surgery must be further examined. Therefore, this study was conducted to determine the relationship between sleep apnoea before CABG and cardiac dysrhythmia after surgery in patients hospitalized at a cardiac surgery intensive care unit (CICU).

## Methods

### Study population

This analytical cross-sectional study was conducted on 192 patients who were admitted to the surgical departments of a specialized cardiac training center in the city of Rasht in the north of Iran for CABG and were included in the study by sequential sampling. The required sample size was determined based on the results of the study by Almeneessier et al. [13], which stated that the chance of heart rhythm disorder in patients with obstructive sleep apnoea (OSA) is 1.91 times that of people without sleep apnoea based on a significance level of 95% and test power of 80%.

The inclusion criteria in this study were: CABG performed with a cardiopulmonary bypass pump, a sinus cardiac rhythm before the operation, no history of rhythm disorder in the past, and no history of diseases such as cancer, stroke, kidney failure and thyroid disorders based on the patient's medical records. The exclusion criteria were: Death or occurrence of ventricular tachycardia, and cardiogenic shock in the operating room or intensive care unit intra- and post-operatively, needing re-intubation, use of an intra-aortic balloon pump during surgery and afterward, and use of a temporary pacemaker during and after CABG.

The instrument used in this study consisted of three sections: (1) Demographic information and disease-related factors, (2) A checklist of heart rhythm disorders after heart surgery related to the disease, and (3) the Berlin questionnaire for obstructive sleep apnoea.

Personal and social information related to the disease included: age, gender, height, weight and education of the person, history of smoking, alcohol and drugs, abdominal and neck circumference and the second checklist includes information such as history of diseases such as diabetes, blood pressure, hyperlipidemia and history of respiratory diseases, preoperative morning tests (hematocrit, creatinine, urea, sodium, potassium and fasting blood sugar and blood cholesterol level), information Related to the examination of the heart, which includes the amount of left ventricular ejection fraction, left ventricular hypertrophy, and right ventricular dysfunction, information related to the operation, including the duration of the heart and lung pump, aortic clamp time, magnesium level after the pump, and information related to suffering from post-rhythm disorder. From the operation that was recorded in the nursing report.

The Berlin questionnaire (BQ) was first introduced in 1998 at a conference in Berlin, Germany, by a group of family physicians and sleep researchers [14]. The present research used the psychometrically assessed Persian version of this tool [11]. This questionnaire contains ten items related to the risk factors of sleep apnoea, which are divided into three subcategories. The first subcategory is related to snoring (with five items), the second subcategory is daytime sleepiness (with three items) and the third subcategory is related to hypertension and obesity (with two items). The overall BQ score was determined based on the responses given to the three subcategories; the scores from the first and second subcategories were taken as positive if revealing frequent symptoms (> 3–4 times/week), whereas the score from the third subcategory was positive if there was a history of hypertension or a BMI > 30 kg/m [2]. Patients were taken as high-risk cases for OSA if they had a positive score on two or more subcategories, while those who did not were scored as low-risk [14].

Due to the fact that the number of available samples was limited, random sampling was not possible, and the researcher was assigned to the cardiac surgery department and gradually entered the samples into the research according to the inclusion criteria. Data were collected from February 2018 to August 2019. In order to collect data, every day, the researcher visited the heart surgery department of the selected hospital where the patients were admitted before CABG, and after selecting the samples, explaining the research objectives, and obtaining informed consent from them, the first and second parts of the tool were completed for each sample (demographic information and variables related to the disease and the BQ). In the second step, after CABG was performed for each of the samples and at the end of their stay in the ICU after heart surgery, the researcher completed the third part of the instrument on the occurrence of heart rhythm disorders by examining the patient’s medical documents. The type of rhythm disorder was also extracted from the nursing reports registered in the ICU and also from the physician reports recorded in the patient’s medical files.

### Statistical analysis

Descriptive and inferential statistics, including the Chi-square test, Fisher's exact test, Mann–Whitney’s U-test, and the backward stepwise logistic regression were used in SPSS software version 22 to analyze the data. The Kolmogorov–Smirnov test was used to check the normal distribution of the parameters. The significance level of the tests was taken as *P* < 0.05.

## Results

In this research, 192 patients were studied. The majority of the patients were male, the mean age was 57.8 ± 7.5 years, and the majority had completed high school. Most of the patients were overweight (44.8%). In terms of patient history,60.9% of the patients under study had hypertension, 39.1% diabetes, and 22.9% had been prescribed inotropes during hospitalization in the ICU after CABG (Table 1).

**Table 1:** The demographic and clinical variables of samples (*n* = 192)

Individual Factors Frequency		Number	Percentage
Age (year)	< 50	24	12.50
	50–59	80	41.67
	> 59	88	45.83
	Mean ± SD	The highest value	The lowest value
	57.79 ± 7.51	70	32
BMI	Normal	47	24.48
	Overweight	86	44.79
	Obese	59	30 .73
	SD ± Mean	The highest value	The lowest value
	27.26 ± 4.08	37	19
Sex	Female	55	28.65
	Male	137	71.35
Education	Illiterate	51	26.56
	High school diploma	130	67.71
	University education	11	5.73
Cigarette smoking	Yes	69	35.94
	No	123	64.06
Alcohol use	Yes	12	6.25
	No	180	93.75
Drug addiction	Yes	36	18.75
	No	156	81.25
Hypertension history	Yes	117	60.94
	No	75	39.06
Diabetes history	Yes	75	39.06
	No	117	60.94
Respiratory diseases history	Yes	3	1.56
	No	189	98.44
Left ventricular hypertrophy	Yes	22	11.46
	No	170	88.54
Right ventricular disorder	Yes	4	2.08
	No	188	97.92
Inotrope administration in the ICU after CABG	Yes	44	22.92
	No	148	77.08

**Fig. 1:**
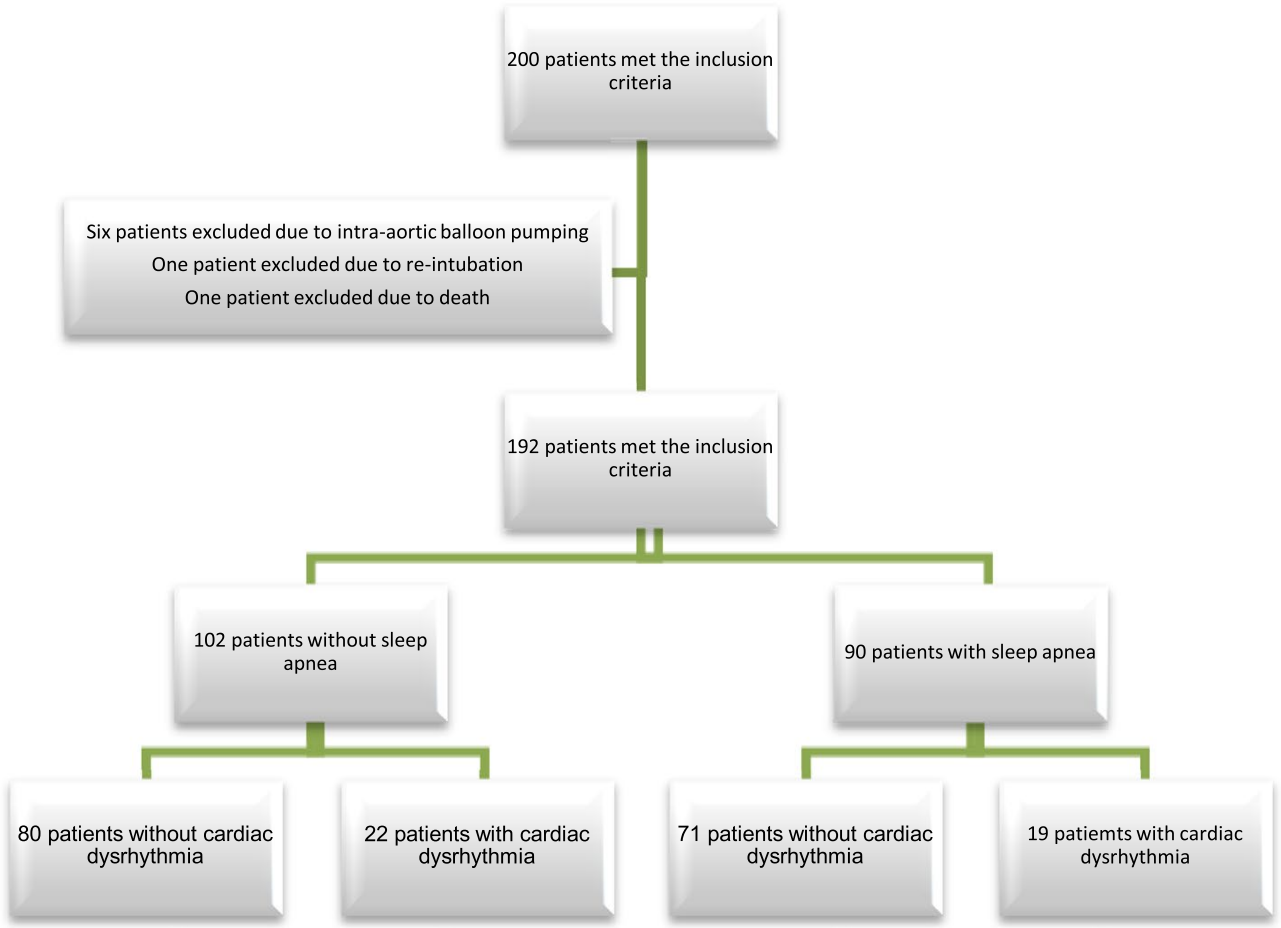
Study flow chart. coronary artery bypass graft surgery and sleep apnea

**Fig. 2:**
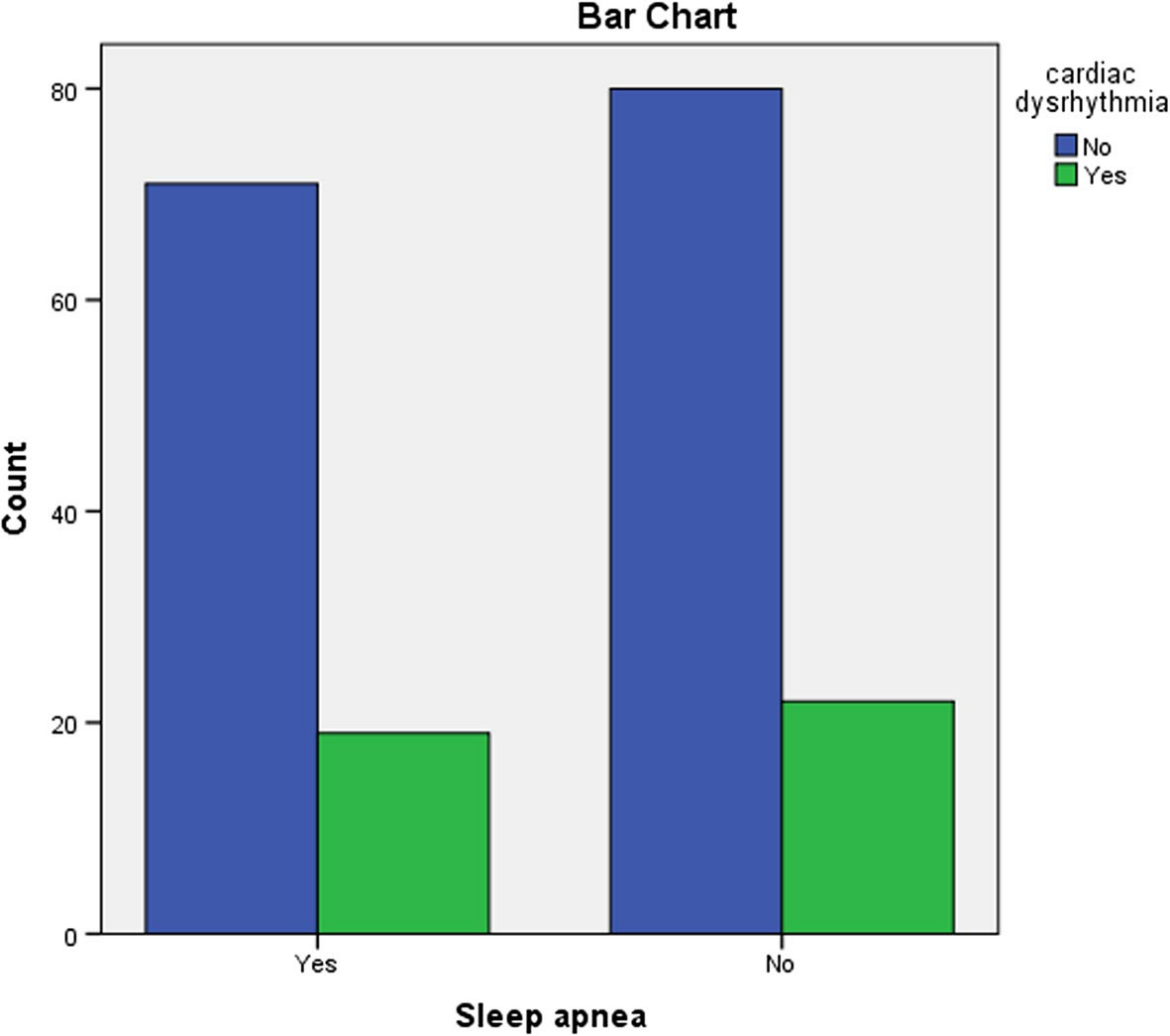
Distribution of heart rhythm disorder after coronary artery bypass graft surgery in patients with and without sleep apnoea

The results showed that 90 of the samples (46.9%) had sleep apnoea, thus confirming that about half of the samples with coronary artery disease had a history of sleep apnoea in this study (Figs. 1 and 2). History of sleep apnoea was significant in relation to three variables: Sex (*P* < 0.001), BMI (*P* < 0.001) and smoking (*P* < 0.001). The frequency of sleep apnoea was higher in the studied women than the men (67.27% of the women compared to 38.7% of the men). Comparing the two groups with sleep apnoea and the group without sleep apnoea showed that blood sugar (*P* = 0.041), haemoglobin (*P* = 0.01), abdominal circumference (*P* < 0.001), and left ventricular ejection fraction (*P* = 0.002) differed significantly between the two groups, as the mean blood sugar, haemoglobin, and abdominal circumference were higher in people with sleep apnoea than those without. Also, 64.9% of the patients with hypertension had sleep apnoea while only 18.7% of those without hypertension had sleep apnoea (*P* < 0.001). Inotrope administration in the ICU after CABG was more frequent in the patients with a history of sleep apnoea (63.6%) compared to the group without such a history (41.9%).

**Fig. 3:**
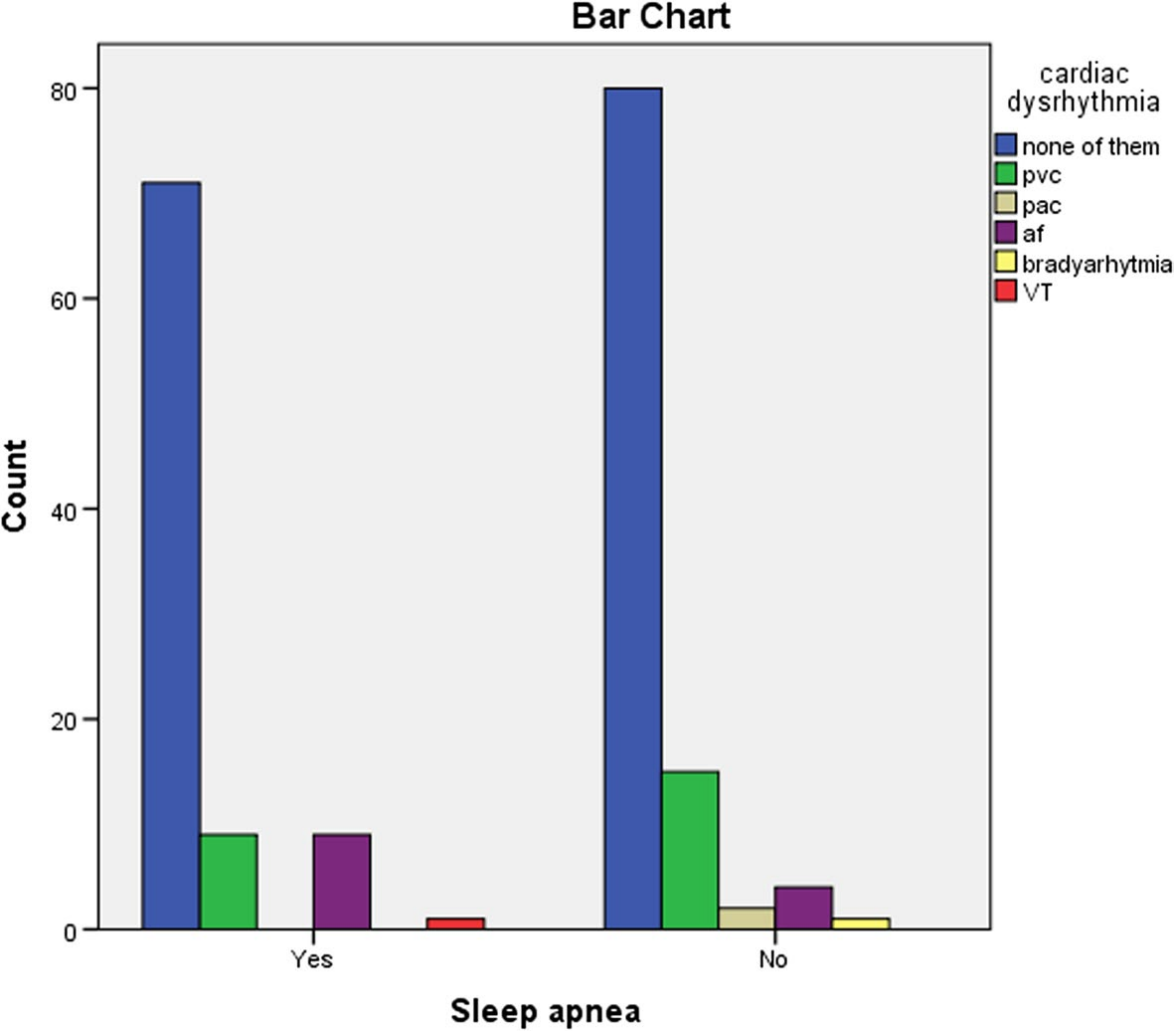
Distribution of heart rhythm disorders after coronary artery bypass graft surgery in patients with and without sleep apnoea

In terms of the frequency of cardiac dysrhythmia after CABG, 21.35% of the samples had dysrhythmia, and the highest percentage of rhythm disorder observed was related to premature ventricular contraction (PVC), atrial fibrillation (AF), and premature atrial contraction (PAC), occurring in 12.5%, 8.6% and 1.04% of the samples, respectively. Also, one patient (0.5%) had bradyarrhythmia, and one patient had ventricular tachycardia (VT) (Fig. 3).

In the multivariate analysis, the logistic regression model was used to investigate the relationship between sleep apnoea and cardiac dysrhythmia after CABG by controlling the effects of the intervening and individual variables. Thus, for the univariate analysis, all the variables with a significance level less than 0.25 were included in the initial logistic regression model. The four variables of smoking history, duration of aortic cross-clamping, hypertension history, and respiratory disease remained in the final model, which was developed by the backward LR method, with only smoking history predicting the occurrence of cardiac dysrhythmia (*P* = 0.005, CI 95%: 1.386–6.566, OR = 3.017). Patients with a history of smoking had a 3.017 higher chance of heart rhythm disorder than non-smokers (Table 2). In the multiple analysis, the relationship between sleep apnoea and cardiac dysrhythmia after CABG was not significant; therefore, sleep apnoea was not considered a predictor of cardiac dysrhythmia after CABG.

**Table 2:** The regression coefficients and odds ratio of the variables related to cardiac dysrhythmia

Variables		Beta	Standard error	*p*	Odds Ratio	95% CI for OR	
						Lower	Upper
Final model	Cigarette smoking	1.104	0.397	0.005	3.017	1.386	6.566
	Duration of aortic clamp	0.025	0.015	0.093	1.025	996.0	1.055
	Hypertension history	-0.678	0.413	0.101	0.508	0.226	1.140
	Respiratory diseases history	-2.225	1.254	0.076	0.108	0.009	1.262
	Constant	4.771	2.843	0.093	118.063		

## Discussion

The relationship between sleep apnoea and cardiac dysrhythmia after CABG and its predictive factors were investigated in this study. The results showed that about half of the samples who had undergone CABG for coronary artery disease suffered from sleep apnoea. The results of some studies have also confirmed that sleep apnoea is common in patients with coronary artery disease [15–17]. It seems that the disturbance in blood supply in coronary artery disease and the insufficient oxygen reaching different organs of the body, including the respiratory system, contributes to the occurrence of sleep apnoea. In addition, the majority of the samples in this research were overweight and obese, and some studies have found a link between overweight and sleep apnoea [7, 18]. The prevalence of sleep apnoea in the study subjects can be explained by noting the possibility of decreased blood supply to various tissues in coronary artery disease and also the overweight and obesity levels. In a review study by Laczay and. Faulx, the relationship between obstructive apnoea and rhythm disorders from epidemiology and pathophysiology and the relationship between obstructive sleep apnea (OSA) and atrial fibrillation have been discussed [19].

The frequency of sleep apnoea differed significantly based on gender, BMI, and smoking, and was higher in women than in men. This finding is in line with the results of Bruno's study, in which women suffered from sleep apnoea more than men [5]. Nonetheless, many other studies have reported different results [20–22] and shown that men suffer more from sleep apnoea than women. In the present study, the number of overweight or obese women was higher than the number of overweight or obese men, and this difference may have influenced the results. Our finding is similar to many studies [7, 13, 22–24]. The results of this research showed that the frequency of sleep apnoea is higher in smokers than non-smokers. Several studies have revealed a relationship between history of smoking and the incidence of sleep apnoea [21, 24].

Based on the results, quantitative variables related to the disease, such as blood sugar, hemoglobin, abdominal circumference, and left ventricular ejection fraction, differed significantly between the two groups with and without sleep apnoea. That is, the mean blood sugar, haemoglobin, and abdominal circumference were higher in the samples with sleep apnoea than those without. Several studies have demonstrated a similar link between the mean blood sugar and sleep apnoea [7, 18, 24, 25]. It seems that as blood sugar increases, the small vessels become narrower, which reduces blood supply to the extremities. The reduction in blood supply to the respiratory system might be the cause of sleep apnoea attacks in these patients. Regarding the relationship between abdominal circumference and the frequency of sleep apnoea, the results of Ochoa’s study also showed that sleep apnoea is more prevalent in people with a larger abdominal circumference [20]. To explain the increase in hemoglobin levels in patients with sleep apnoea, we must bear in mind the compensatory mechanism of an increase in hemoglobin levels in response to nocturnal hypoxia. One of the unexpected findings of this study was that the left ventricular ejection fraction was higher in the group with sleep apnoea than the group without, while studies by Uchôa et al. [20], Schmidleitner et al. [18] and Tafelmeier et al. [26] have shown decreased left ventricular ejection fraction with sleep apnoea, which is in contrast to the present findings. The small number of samples with a history of sleep apnoea in this study might have influenced this finding. To explain these findings, it should be noted that the samples in this research underwent echocardiography by different physicians, and the interval between echocardiography and CABG was not the same in all of them. Considering that the left ventricular ejection fraction was not recorded in the angiography report of all the samples, its value was extracted from the echocardiography reports. In the present study, the majority of patients with hypertension had sleep apnoea, which is in line with the results of some other studies [7, 24, 26, 27]. The frequency of sleep apnoea was also higher in diabetic patients than in non-diabetic patients. Many other studies have reported a similar relationship between sleep apnoea and diabetes [7, 18, 24, 25, 28]. In addition, more cardiac inotropes were prescribed to the patients with a history of sleep apnoea in the ICU after heart surgery compared to those without such a history. This finding is in line with the results reported by Tafelmeier et al. [26]. Inspiratory effort creates more negative pressure in the chest in sleep apnoea, thus causing hemodynamic effects and putting pressure on the heart. The negative pressure of the chest, which is normally -8 cm of water, can increase to -30 cm of water during a respiratory pause, thus raising the venous return and ultimately increasing the afterload. Given the imbalance between the oxygen supply and demand in the left ventricular muscle as a result of the higher need for oxygen due to the increase in afterload, there is more need for prescribing cardiac inotropes [9].

About 23% of the studied samples had cardiac dysrhythmia after CABG, and the highest percentage was related to PVC, AF, PAC, bradyarrhythmia, and VT. The results of this study are in line with the study by Schmidleitner et al. [18], which showed that ventricular rhythm disorder after coronary artery bypass surgery is a major problem that could cause sudden death. The present findings are also in line with the results of Bruno's study, which showed that 50% of the subjects suffered from atrial fibrillation after surgery [5]. The patients in the present study had all undergone open-heart surgery, during which the surgeon was forced to manipulate the heart tissue in order to perform cannulation and coronary artery transplantation. This tissue manipulation may activate the deviant pathways and cause rhythm disturbances. In addition, electrolyte disorders caused by the patient being attached to a heart–lung machine and the tissue ischemia caused by surgery may have been effective in activating these deviant pathways. In their study, Rezaei et al. mention that the development of AF arrhythmia after surgery is a multifactorial phenomenon and some of the main influential pathogens include inflammatory pathways, oxidative stress, and autonomic dysfunction. In addition, several predisposing factors lead to the development of AF, including pre-, intra-, and post-operative factors such as age, previous history of major cardiovascular risk factors, and ischemia–reperfusion injury during surgery [29].

In the multivariate analysis, the logistic regression model was used to investigate the relationship between sleep apnoea and cardiac dysrhythmia after controlling the effects of the intervening and individual variables. In the final model, smoking history was the only variable that predicted the occurrence of cardiac dysrhythmia, as the smokers had a higher chance of cardiac dysrhythmia than the non-smokers, which is in line with the results of many other studies [30–32]. Nicotine consumption can lead to atrial fibrosis, which can increase the probability of cardiac dysrhythmia. In addition, long-term smoking causes hypoxia of the heart tissue and leads to structural changes, especially changes in tissue blood supply to the heart, and contributes to cardiac dysrhythmias. In a multiple analysis, the relationship between sleep apnoea and cardiac dysrhythmia was not significant and the results could not confirm the role of sleep apnoea in the occurrence of cardiac dysrhythmia after CABG.

The main limitation of the study was the use of a questionnaire to investigate sleep apnea, which the research samples responded to, and the lack of access to polysomnography in the correct diagnosis of sleep apnea, and the researchers themselves did not observe the occurrence of sleep apnea, and therefore, it is possible, what that the patient described was actually not apnea. Another limitation of this study was the use of medical records to investigate dysrhythmias that may have been associated with errors, and the small number of samples was another limitation of the study that may affect the research results. Therefore, it is suggested to conduct more studies with a larger sample size.

## Conclusion

The results of this study showed that there is no statistically significant relationship between sleep apnoea and the occurrence of rhythm disorders after CABG. Also, the only variable related to the occurrence of cardiac dysrhythmia after CABG was history of smoking; considering that smokers are more prone to heart rhythm disorder after heart surgery, the researchers recommend cohort or case–control studies to investigate the effect of smoking history on rhythm disorders after CABG.

## Data Availability

The datasets used and analyzed during the current study available from the corresponding author on reasonable request.
